# The role of interviewing in endocrine practice

**DOI:** 10.1007/s40618-025-02565-w

**Published:** 2025-03-29

**Authors:** N. Sonino, G. A. Fava, D. C. Aron, Jenny Guidi

**Affiliations:** 1 Department of Statistical Sciences, University of Padova, Padova, Italy; 2 Department of Psychiatry, State University of New York at Buffalo, Buffalo, NY USA; 3 Case Western Reserve University, Cleveland, OH USA; 4 Department of Psychology “Renzo Canestrari”, University of Bologna, Viale Berti Pichat 5, Bologna, 40127 Italy

**Keywords:** Interviewing, Allostatic load, Endocrine disease, Psychosocial factors, Quality of life

## Abstract

**Background:**

Interviewing is a basic, yet neglected clinical method that allows to understand how a person feels and what are the presenting complaints, obtain medical history, assess personal attitudes and behavior related to health and disease. In the endocrine setting it provides the patient with information about diagnosis, prognosis and treatment, and establishes a therapeutic relationship that is crucial for shared decision making and self-management. However, the value of this clinical skill is threatened by time pressures and emphasis on technology. Current health care trends privilege expensive tests and procedures and tag the time devoted to interaction with the patient as lacking cost-effectiveness. Instead, the time spent to enquire about problems and life setting may actually help to avoid further testing, procedures and referrals.

**Methods:**

The aim of this paper is to provide an overview of optimal use of interviewing in clinical endocrinology.

**Results:**

The basic principles of the art of interviewing are described, particularly as to medical diagnosis and history, health attitudes and behavior (including lifestyle), patient’s experience of symptoms and quality of life, allostatic load and psychological distress.

**Conclusions:**

Assessment by interviewing may indeed offer a characterization of the person’s psychosocial environment that is missing from current formulations. It may shed light on a number of clinical issues, such as interpretation by the endocrinologist of abnormal hormone values that lack explanation, difficulties in coping with the various phases of illness, maladaptive illness behavior, presence of residual symptoms.

## Introduction

Clinical interviewing is the basic method to understand how a person feels and what are the presenting complaints, obtain medical history, evaluate personal attitudes and behavior related to health and disease. It gives the patient information about diagnosis, prognosis and treatment, and establishes a bond between patient and physician that is crucial for shared decision making and self-management [1]. The value of this basic skill is often underestimated and threatened by time pressures and emphasis on technology [1]. In clinical endocrinology in particular there is often the tendency to rely exclusively on “hard data”, preferably expressed in the dimensional numbers of laboratory measurements, excluding “soft information” such as the individual perception of symptoms and the level of disability [2]. The hegemony of the biomedical model of disease, as exemplified by “precision medicine”, is however in striking contrast with the emerging awareness of the importance of psychosocial determinants of health [3, 4] and patient experiences [2]. Such awareness has become prominent in the areas of obesity and diabetes [5–7], but it is still insufficiently appraised in clinical endocrinology at large [2].

The aim of this paper is to provide an overview of the role of interviewing in clinical endocrinology. We will examine its basic principles, discuss its importance in the various phases of disease, and describe its therapeutic implications.

## Methods

References for this review were identified through searches from PubMed for articles published from inception to December 2024, with the terms “interviewing” or “interview” or “medical history” and “endocrine”, “Cushing”, “Addison”, “aldosteronism”, “thyroid”, “hyperparathyroidism”, “hyperprolactinemia”, “acromegaly”, “polycystic ovary syndrome”. Articles in English involving human subjects resulting from these searches were reviewed. A manual search of the reference lists of the selected articles was performed in order to identify additional relevant papers.

## Basic principles

Interviewing consists of essentially two forms of questions. One is based on open-ended type of questioning, which allows patients to speak freely about their condition, with questions such as “how are you?” or “how do you feel?” [1, 8]. The other method involves closed-ended questions, such as “do you feel any pain?” or “do you feel tired?” that often follow an interrogative “yes/no” questioning that is in line with electronic medical records. The two strategies are generally mixed in clinical practice. An initial open-ended question may be followed by closed-ended questions based on the patient’s initial response. However, care should be taken in not interrupting the patient too early (within a minute) in the course of the interview, as it is often found to be the case [9].

Clinical interviewing is not only based on verbal exchanges and patient’s answers. The course of the interview may be channeled toward directions which are suggested by observation of the patient and findings during physical examination. Non-verbal communications (e.g., gestures, posture, facial expression, way of speaking) may provide additional sources of inquiry [1, 8, 9]. Such interactional setting requires close attention and engagement and is unlikely to occur with an interviewer who only watches the computer screen instead of the patient [8].

Clinical reasoning goes through a series of “transfer stations”, where potential connections between presenting symptoms and pathophysiological processes are considered [10]. Transfer stations are a pause for verification, or change to another direction. Clinicians should think ahead and have the next question ready while still listening to the answer to the previous question, to keep the flow of the interview [1]. Certain signs can only be discovered if they are suspected and searched for [11]. Symptoms and comorbidities that at first glance may appear to be unrelated and scattered among different organ systems may then reveal unifying features.

Even though there are many questionnaires that may provide data for medical history [9], and patient-reported outcome measures appear to be of value [12], interviewing is the key instrument for collecting information on the present and past state of a person.

## Clinical domains

Collecting information regarding the general condition of the patient, as well as signs and symptoms and medical history, are the universally recognized goals of the medical interview [1]. There is growing awareness of the importance of assessing psychosocial, cultural, and contextual factors that make individuals susceptible to disease, resilient when disease arises, and variably responsive to treatment [4–7, 13]. In endocrine practice, there are several areas where clinical interviewing displays its importance.

### Medical diagnosis and history

In general medical practice the majority of final diagnoses can be made on the basis of patient’s history [9]. A careful collection of signs and symptoms is of paramount value for orienting laboratory investigations [14]. Appraisal of the symptomatology and the time of appearance are important, since symptoms of endocrine disorders often develop insidiously and are non-specific (e.g., fatigue and weight changes). A similar symptom can occur both in disorders of endocrine hypofunction and hyperfunction (e.g., apathy in both hypothyroidism and apathetic hyperthyroidism) [15]. Among the challenges faced by clinical endocrinologists where excellence in history taking can make a positive difference is the case of the patients with subclinical hypothyroidism. Such patients may have non-specific symptoms that are also highly prevalent in the general population. Causal attribution of symptoms may be difficult [16]. Even more problematic for both practitioner and patient is the fact that in 5–10% of cases, symptoms persist even after treatment with thyroxine that normalizes TSH levels. The interactive process of history taking can pre-emptively address causes of treatment dissatisfaction by understanding the patient’s experience and managing expectations, particularly since some patients may end up in the groups with medically unexplained symptoms [17, 18]. Within the wide spectrum of functional adrenal disorders, examples where accurate anamnestic inquiring is crucial are arterial hypertension as to secondary endocrine causes [19] and the case of mood disorders in patients with endogenous hypercortisolism [20].

The high prevalence of medication and food interference with laboratory tests and hormone absorption makes essential to collect all relevant information [21, 22]. Medication history should not be limited to prescribed medications, but extended to over-the-counter drugs, nutrient and supplements because they can influence both endocrine function and laboratory results. Their use may be initially denied by the patient, yet the expert clinician may obtain appropriate information by reiterating the question at some later point in time during the visit, when a satisfactory relationship has been established.

Treatment history appears to be particularly complex in view of the frequency of polypharmacy [23] and requires accurate exploration, a process that can be compromised if an insufficient amount of time is allotted.

Baum [24] emphasizes the need for an endocrinologist to communicate in an effective fashion and to provide an adequate patient education. For instance, in primary adrenal insufficiency [25] and in osteoporosis [26] positive effects of patient education on the management of disease have been reported.

### Health attitudes and behavior

Individual attitudes and behavior related to health and disease are major components of clinical encounters [27]. These factors shape lifestyle, presentation of symptoms, access to patient care, interactions between patients and physicians, adherence to medical advice, response to treatment. Health attitudes and behavior may range from anxiety and worry about illness, to various forms of denial, such as delay in seeking care, and lack of adherence to treatment [28, 29]. Habits such as a healthy diet, regular physical activity, safety practices, adequate sleep and refraining from smoking and substance use, are increasingly recognized as major factors in enhancing health and reducing the risks associated with disease [27]. The ways in which individuals experience, perceive, evaluate and respond to their own health status, play a major role in the delay in seeking medical help and in treatment adherence [30]. In practice, many endocrine disorders are diagnosed after a considerable delay from the onset of symptoms and such delay may entail profound psychological effects on the patient, as in the case of Cushing’s syndrome [31] and in polycystic ovary syndrome [32]. Listening to the patient’s beliefs on the illness and its treatment, with an open-ended style of interviewing, may identify inappropriate expectations and convictions that may yield health-damaging behaviors [27]. Table 1 displays specific questions that may be helpful during the clinical interview and that may be supplemented by questionnaires.

**Table 1:** Questions that may help assessing health attitudes and behavior during the clinical interview

If you are suffering from common symptoms (e.g., a cold, headache), do you fear they may develop into a serious illness?
Are you afraid of suffering from a specific disease, even though your doctor and the laboratory examinations have excluded any specific medical illness?
Do you often worry that you may have a serious illness that your doctors have not been able to diagnose?
Have you ever experienced these fears for at least 6 months?
Do you keep worrying about your health after the doctor gives you an appropriate medical reassurance explaining that you don’t have any illness and you are healthy?
Have you ever neglected to bring to your doctor’s attention serious symptoms?
If the doctor tells you that you have a disorder and prescribes you drugs, a suitable diet or an appropriate physical activity, do you follow the medical advice?

### Patient experience of symptoms and quality of life

There is increasing awareness of the importance of prioritizing patient experiences of symptoms and quality of life in diabetes [7] and endocrinology [33, 34]. Indeed, the subjective perception of health status is as valid as that of the clinician in evaluating outcomes [12]. There may be discrepancies between the clinical state of an individual according to objective measurements and the unique personal perception of health, as it may occur in the management of adrenal incidentalomas where key questions must be addressed in dialogue with the patient about need for further testing, nature of the tumor, benefits and disadvantages of adrenalectomy [35].

Quality of life is variably compromised in different endocrine diseases and does not necessarily improve as expected upon successful treatment and normalization of hormone values [36–41]. For example, there are patients who report persistent symptoms despite achieving normal thyroid levels upon thyroid replacement [42]. Resilience (the capacity to overcome adversities and stress while maintaining normal and physical functioning) has been found to modulate quality of life in neuroendocrine neoplasms [43]. Psychological well-being associated with the presence of positive affect may modulate the individual reaction to illness in direction of resilience and euthymia [44]. The concept of euthymia means achieving flexibility of the response capacity of the individual in dealing with stress and the challenges of medical disorders [44]. Euthymia has been seldom addressed in clinical endocrinology [43], but it is attracting interest in the domain of diabetes [45, 46].

### Allostatic load

Exposure to acute and chronic stress and other environmental factors can disrupt optimal neuroendocrine activity [4–6, 47]. Allostatic load refers to the cumulative burden of both chronic stress and stressful life events [48, 49]. When environmental challenges exceed the individual ability to cope, allostatic overload ensues [49]. Assessment of allostatic load/overload by clinical interview, in addition to biomarkers, offers a characterization of the person’s psychosocial environment [50, 51], that include the social determinants of health. Table 2 reports questions that are geared to the identification of allostatic load/overload, but that also address the life context of the patient. Most of them are derived from the PsychoSocial Index [51, 52], a self-rating questionnaire to be filled by the patient at the time of the initial endocrine consultation. In clinical practice settings, it can be simply scanned by the physician, with opportunities for interaction and clarification. The presence of a state of allostatic overload may provide an important clue for interpreting abnormalities in hormone values that lack other explanations and that may be affected by stressful life situations, like increased prolactin levels [50]. Appraisal of life stress may have implications for clinical decisions in the course of disease and in the case of unexplained somatic symptoms or delayed recovery [50]. Finally, higher allostatic load may predict poorer health outcomes and is associated with marked psychological distress [49, 50].

**Table 2:** Questions that may help determining a state of allostatic load/overload during the clinical interview

In the last 12 months, did a family member or a close friend die? Did you separate or divorce from your partner? Did you change job? Move?
Did you have severe economic difficulties? Did you have legal problems? Were you without job? Have you been a victim of bullying, stalking, racism or severe interpersonal pressure? Did you have problems with your spouse /partner or other family members? Did you feel tension at home? Has one family member been seriously ill?
Have you had the feeling that life is asking you too much?
Did it happen to take a long time to fall asleep? Did you wake up many times during the night? Did you wake up too early and could not go back to sleep?
Did you feel tired, without energy? Did you feel a sense of instability, dizziness? Did you feel nervous or anxious? Did you feel irritable? Did you feel sad or depressed? Did you feel demoralized?
Did you have problems or difficulties at work, at home or in relationships with other people?
Did you feel overwhelmed by the demands of everyday life?

### Psychological distress

Endocrine disorders may be associated with a wide range of psychological symptoms [2, 20, 41, 42]. At times, such symptoms reach the level of psychiatric illness, mainly mood and anxiety disorders, and may precede other manifestations of the disease and/or are early indicators of its relapse [2, 20]. It is thus important to inquire about depressive symptoms and anxiety. Table 3 lists questions that may be helpful in eliciting the presence of a major depressive disorder, that is depressed mood associated with other symptoms (loss of interest or pleasure, appetite changes, sleep disturbances, psychomotor retardation or agitation, fatigue, feelings of worthlessness and guilt, diminished ability to think or concentrate, suicidal thoughts). The questions are derived from the Clinical Interview for Depression [53]. Symptoms of depression should be looked upon particularly in Cushing’s syndrome, hyperthyroidism, hypothyroidism, hyperparathyroidism and hyperprolactinemia. They could be prodromal and create difficulties in differential diagnosis. Anxiety disorders are common in most endocrine conditions [2] and in primary aldosteronism they may appear as isolated affective disorder [54]. Depressive and anxiety symptoms may persist after treatment of the disorders [2, 20].

**Table 3:** Questions that may help assessing psychological distress during the clinical interview

In the last month, have you felt depressed, moody, downhearted, dejected, sad, blue, about to cry? How often? How long does it last? How bad is it? At what time of the day do you feel worse?
How much has feeling bad affected your capacity to do your work and other activities? Have you lost your interest in most things?
Does it take a long time to fall asleep? Do you wake up many times during the night? Do you wake up too early and could not get back to sleep? Have you been sleeping more than usual?
Have you been feeling nervous, anxious, frightened, scared and panicky? Have you found it hard to relax? Have you had a feeling of dread, as though something terrible were about to happen?
Are there any particular situations that tend to make you feel anxious, or lead you to avoid them (e.g., leaving home alone, going into public or crowded places, driving, traveling by public transport, social situations)?
Do you feel demoralized or discouraged because there is a pressing problem you feel unable to cope with or because you are not adequately supported by friends and/or relatives? Do you feel you have failed to meet your own expectations or those of other people (concerning your work, family, social and/or economic status)? Have you been feeling this way for longer than a month?
Have you been getting irritated with people more easily recently? Have you been losing your temper? Do you have uncontrollable verbal or behavioral outbursts? How often? After that, do you still feel bad?

In many cases, however, psychiatric nosography fails to capture psychological distress, and this can only be identified by assessing its subclinical forms, such as demoralization and irritable mood. Demoralization is characterized by the patient’s consciousness of having failed to meet his or her own expectations or those of others or being unable to cope with some pressing problems [55]. In the various phases of endocrine diseases there are important sources of demoralization, such as the long interval from appearance of the first symptoms to establishment of a proper diagnosis, the period for endocrine work-up which may be lengthy and fatiguing, the long time required to recover after surgery or radiotherapy [2, 20, 55]. Irritability has been associated with several endocrine disorders [2], particularly in Graves’ disease [56] and hyperprolactinemia [57]. It becomes a condition worthy of clinical attention when it is characterized by a prolonged and generalized state, with difficult control over temper, or by angry-explosive attacks [58]. Demoralization and irritable mood may persist after normalization of endocrine parameters [36]. Table 3 lists some questions to recognize the presence of demoralization and irritable mood [53, 55, 58].

## Clinical implications

Current health care trends do privilege expensive tests and procedures and tag the time devoted to patient interaction as lacking cost-effectiveness. Unfortunately, time pressure for patient evaluation does not allow an adequate appraisal of comorbidities and medication history, that are major issues in endocrine practice. On the contrary, the time spent to enquire about problems and life settings may be cost-effective, saving on unnecessary testing, procedures and referrals [1, 9]. Thus, the complementary roles of clinical interviewing and advanced diagnostic tools should be acknowledged. Accordingly, physicians should make any effort to obtain an adequate time for interviewing in both public and private sectors. Indeed, in a reimbursement system, clinical interview should become the best paid procedure, since it is an extraordinary investigative technique. Such general considerations particularly apply to clinical endocrinology.

Listening to the patient’s life context and emotions and exploring his/her beliefs on the illness and its treatment, with an open-ended style of interviewing, may disclose the patient’s experience of illness. Specific questions (Tables 1, 2 and 3) may follow. Additional key information may derive from self-rated and observer-rated instruments, that pertain to the domain of clinimetrics, the science of clinical measurements [59, 60].

A comprehensive view may provide crucial information for understanding and managing several issues: health-damaging behavior, such as unhealthy lifestyle; levels of disability and/or compromised quality of life in relation to what is expected by disease status; insufficient participation in self-management and/or rehabilitation; lack of treatment adherence; failure to resume a healthy role after convalescence; illness denial. Comorbidity, that is usually considered as a confounder of the single-diagnosis approach, must become a leading direction and target, in line with the major transformations in health care needs that have occurred in the past decades [61]. For instance, after discharge from the hospital, patients face a transient period of general vulnerability to disease and an elevated risk of adverse events (including hospital readmission and mortality), sometimes presenting with the features of post-hospital syndrome [62]. This syndrome could also be viewed as a consequence of allostatic overload (sleep disruption, mobility impairment, pain, fears, psychological distress) [55].

Some areas may be illustrative of the value that an extended time dedicated to clinical interviewing may yield.

### The partnership paradigm

There is increasing emphasis on the importance of the participation of patients in their own management [7, 9, 27, 33, 34]. The partnership paradigm includes both collaborative care, a patient-physician relationship in which physicians and patients go through all phases of the clinical process, share treatment preferences, and reach an agreement on treatment choices, and self-management, that is concerned with the participation of patients in their therapeutic plan. These aspects have been recently highlighted as essential part in the care of adrenal incidentalomas [35]. In many cases, problem areas may be amenable to improvement through provision of medical information and adequate explanation. This is particularly important in individuals with limited health literacy, who would otherwise be prone to worse self-management, lower use of preventive services, and higher hospitalization rates [63]. Unhealthy diet, overweight, lack of physical activity, alcohol consumption, smoking habits often play a major role in the pathogenesis, course and response to treatment of endocrine disorders [4, 7, 50]. The metabolic syndrome is often a consequence of harmful lifestyles, frequently associated with allostatic load, in settings of poverty and social inequalities [64]. Motivating people to make beneficial changes in their behavior is now regarded as a current health care challenge. Lifestyle medicine has emerged as a major component of treatment of medical disorders [65, 66]. Evidence from controlled trials indicates that programs teaching problem-solving skills and self-management may improve clinical outcomes and reduce costs [65–67]. However, these achievements require for the patient a shift from the role of passive consumer to that of health producer and for the organization of care a major time investment on patient-doctor communication [67].

An additional issue pointing to the importance of clinical interviewing is represented by the fact that people commonly perform online health searches before seeing a physician [68]. Lack of opportunity of discussing relevant health information has been found to result in dissatisfaction and impaired patient-clinician relationship [68].

### Clinical interviewing as part of treatment

In the past two decades, people with medical disorders have made clearer a wish to stay on the top of managing their own illness and make healthy choices. Such struggles have been particularly impressive in the field of diabetes [7]. Motivational interviewing is a technique geared to facilitate lifestyle changes, that has been successfully applied to diabetes, as well as to other medical disorders, to induce behavioral changes in areas such as eating habits and physical activity, and to achieve an effective use of medications and medical care [69, 70].

Clinical interviewing may not be simply motivational, but it may entail therapeutic connotations. Harvey Cushing wrote: “Unconsciously or otherwise all clinicians are psychotherapeutists and undoubtedly he is the more successful with his patients who effectively uses this agency to allay the individual’s morbid depression or doubts, anxiety or fears” [71, p.967]. Indeed, health attitudes and behavior may be responsive to psychotherapeutic management (application of psychological understanding), that should not be confused with formal psychotherapy [27]. Also treatment expectations may influence outcome [72]. If a new treatment is presented as breakthrough cure, its failure sets the stage for the onset of demoralization, a feeling state characterized by subjective incompetence, discouragement, helplessness and hopelessness [55, 72]. Unfulfilled expectations about therapy may be interpreted by patients as an indication that their condition is untreatable, and such conviction may be reinforced if they perceive disillusionment and frustration in the clinician. For instance, patients with hypothyroidism may assume that all the symptoms they experience are due to their thyroid condition and/or to inadequate treatment, while symptoms may have other sources. It is thus important for the endocrinologist to provide explanation with an empathetic approach [24].

When treatment choices are involved, particularly in case of drug therapy, there are different physician’s skills that may enhance patients’ adherence, including a clear explanation of the treatment rationale, information about benefits and potential adverse effects, choice of regimens less complex as possible, and follow-up visits [27].

### Assessing recovery and the need for rehabilitation

In recent years there has been increasing awareness of the unsatisfactory degree of remission that current therapeutic strategies entail in a variety of endocrine disorders [73–78]. There may be different reasons for a delayed or impaired process of recovery, such as persistent alterations of hormone values, hormone replacement that does not fully restore optimal endocrine balance, unrealistic expectations of a quick recovery, the presence of allostatic load, impairment in self-esteem, body image distortion, disruption in interpersonal relationships, social withdrawal [73]. Such factors may hinder work performance and economic deterioration may ensue [75], which then becomes the source of additional allostatic load.

**Fig. 1:**
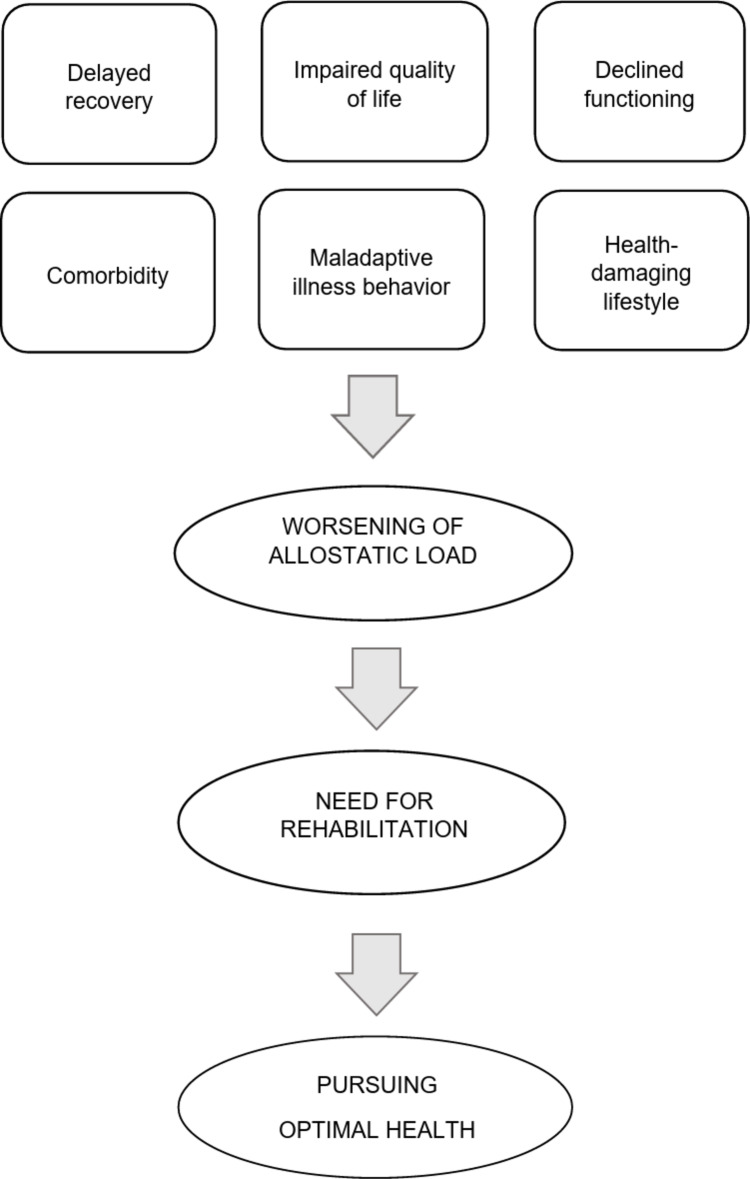
Main indications for rehabilitation in endocrine patients

Clinical interviewing plays a crucial role in the post-treatment phase of endocrine disease and is the main source of information for establishing whether incomplete recovery warrants a rehabilitation process [79, 80]. An adequate time should be allocated to assessment, introducing some innovative features (Table 4) compared to a traditional endocrine consultation. Rehabilitation of endocrine patients should be the sum of activities required to ensure them the best physical, mental and social conditions so that they may progress to an optimal state of health [79]. Figure 1 outlines the main indications for rehabilitation in endocrinology: delayed recovery after surgical intervention or radiotherapy; impaired quality of life with discrepancies between endocrine status and current functioning; decline in physical and social functioning; persistence or onset of important comorbidity with particular reference to psychological disturbances; maladaptive illness behavior, that includes poor adherence to treatment and difficulties in self-management; health damaging lifestyle and risk behavior. These clinical situations may build into a state of allostatic load, that should be addressed using individualized strategies [50].

**Table 4:** Endocrine consultation in the post-treatment phase

Update of history, including psychosocial aspects
Physical examination
Appraisal of the present situation, based on all findings, with special reference to quality of life, impaired functioning and illness behavior
Explanation and education
Discussion of treatment issues, including compliance
Prescriptions
Letter to the referring physician, with prescriptions about lifestyle modifications, psychological support and rehabilitation measures, as needed

A major focus should be on improving self-management and compliance, that may be hindered by prejudice and wrong beliefs about the prescribed medication or replacement hormone treatment. Physical impairments and lifestyle issues may be addressed by specific specialists, if needed. Psychiatric consultation and/or brief individual psychotherapy may be indicated in selected cases. Group therapy of patients sharing similar disorders (e.g., pituitary disease) might also be of help. Emotional sharing, reassurance, provision of information, and planning for the future would encourage the patient to think in terms of adaptive coping, rather than brood about the past [80].

## Conclusions

Interviewing is a major clinical skill and its quality in endocrinology determines the quality of data that are collected [11] and, eventually, of assessment and treatment. Thus, interviewing deserves more attention in educational training and more space in clinical encounters than is currently receiving. Its opening to the psychosocial determinants of health in endocrine disease allows to view illness within the interaction between the person and the environment (Tables 1, 2, 3 and 4), that has a lot to do with the increasing rate of metabolic abnormalities (obesity, diabetes, metabolic syndrome) worldwide [4]. The clinical interview may help to demarcate substantial differences among patients who otherwise seem deceptively similar because they share the same medical diagnosis [1].

Dedicating more time to the clinical interaction is also important for physicians’ well-being. In a setting where there is less and less time for talking with the patients, the physician may feel overwhelmed by clinical duties and less tolerant to the unavoidable nuisances of the profession [81]. Indeed, positive relationships with patients and purpose in life are a major source of gratification and meaning for physicians.
